# Long-term outcomes of sacral and sacrococcygeal chordomas: a 30-year single-center experience comparing 3 treatment modalities

**DOI:** 10.1016/j.spinee.2026.06.009

**Published:** 2026-06-09

**Authors:** Takashi Hirase, Jessica A. Lavery, Yoshiya Yamada, Mark H. Bilsky, Max Vaynrub, Patrick J. Boland

**Affiliations:** a Department of Surgery, Memorial Sloan Kettering Cancer Center, 1275 York Avenue, New York, NY 10065, USA; b Department of Epidemiology and Biostatistics, Memorial Sloan Kettering Cancer Center, 1275 York Avenue, New York, NY 10065, USA; c Department of Radiation Oncology, Memorial Sloan Kettering Cancer Center, 1275 York Avenue, New York, NY 10065, USA; d Department of Neurosurgery, Memorial Sloan Kettering Cancer Center, 1275 York Avenue, New York, NY 10065, USA

**Keywords:** Local recurrence, Overall survival, Radiotherapy, Sacral chordoma, Sacrococcygeal chordoma, Surgical resection

## Abstract

**BACKGROUND CONTEXT:**

Sacral and sacrococcygeal chordomas present surgical and clinical challenges distinct from those of chordomas in the mobile spine. Over the last 30 years, 3 primary treatment modalities have emerged: wide surgical resection alone, surgical resection with radiotherapy (RT), and increasingly definitive RT. However, no studies have directly compared outcomes among the 3 treatment methods.

**PURPOSE:**

To compare local recurrence (LR), overall survival (OS), perioperative outcomes, and complications among patients with primary solitary sacral or sacrococcygeal chordomas treated with wide surgical resection alone, surgical resection with RT, or definitive RT.

**STUDY DESIGN:**

Retrospective cohort study.

**PATIENT SAMPLE:**

One hundred thirteen patients with sacral or sacrococcygeal chordoma who were treated at a single institution between January 1994 and February 2024.

**OUTCOME MEASURES:**

Primary outcome measures were LR and OS. Secondary outcome measures were 30-day and long-term complications.

**METHODS:**

Differences in LR and OS among treatment groups were analyzed using multivariable Cox proportional hazard models weighted by inverse probability of treatment weights. Rates of 30-day complications were compared using chi-squared tests; incidence of complications at any time following treatment was modeled using a negative-binomial regression model.

**RESULTS:**

Median follow-up was 7.6 years (interquartile range, 2.7–11). Fifty-five patients (49%) underwent wide surgical resection alone, 40 (35%) underwent wide surgical resection with neoadjuvant or postoperative adjuvant RT, and 18 (16%) received definitive RT. Negative margins were achieved in 83 patients (87%) undergoing surgery. OS varied significantly across treatment modalities and was shorter among patients who underwent surgical resection alone (hazard ratio [HR], 2.01; 95% confidence interval [CI], 0.96–4.20) or definitive RT (HR, 3.73; 95% CI, 1.15–12.1) compared to patients who underwent surgery with RT. LR was seen in 31 patients and did not differ across treatments in multivariable analysis (p=.3); the HRs for surgery with RT and definitive RT were 0.48 (95% CI, 0.17–1.31) and 1.01 (95% CI, 0.25–4.12), respectively, relative to surgery alone. Among patients who received RT, a higher biologically effective dose was associated with a lower risk of LR (HR, 0.92; 95% CI, 0.86–0.98; p=.01). Complication rates at 30 days differed among the surgery plus RT (85%), surgery alone (55%), and definitive RT (5.6%) groups (p<.001), but total complication rates at final follow-up did not vary significantly across groups.

**CONCLUSIONS:**

Wide surgical resection has been the recommended treatment for solitary primary sacral and sacrococcygeal chordoma. The addition of an RT regimen may improve LR and survival. Although definitive RT demonstrated short-term outcomes comparable to those of surgery alone, longer follow-up is required to determine the durability of response and the late-toxicity profile. Therefore, definitive RT should be considered selectively rather than as a replacement for surgery.

## Introduction

Chordomas are rare malignant neoplasms of notochordal origin and account for 1% to 4% of primary malignancies of bone [1,2]. Although they can occur at any site within the axial skeleton, approximately one-third originate in the sacrum [3]. Sacral and sacrococcygeal chordomas present clinical and surgical challenges that differ substantially from those posed by chordomas of the mobile spine. They are often painless and asymptomatic until reaching a substantial size; the most common presenting symptoms are low back pain and radiculopathy related to the mass effect on sacral nerve roots [4,5]. Depending on the cephalad-most extent of tumor involvement, surgical resection with an attempt to achieve negative margins can lead to significant morbidity, including permanent loss of bowel, bladder, and sexual function [4,5]. Nevertheless, a wide resection is achievable for sacral and sacrococcygeal chordomas (unlike their counterparts in the mobile spine), particularly when the tumors involve the distal sacrum, for which the surgical neurologic and structural sequelae are less severe than those of high sacral tumors.

**Fig. 1.**
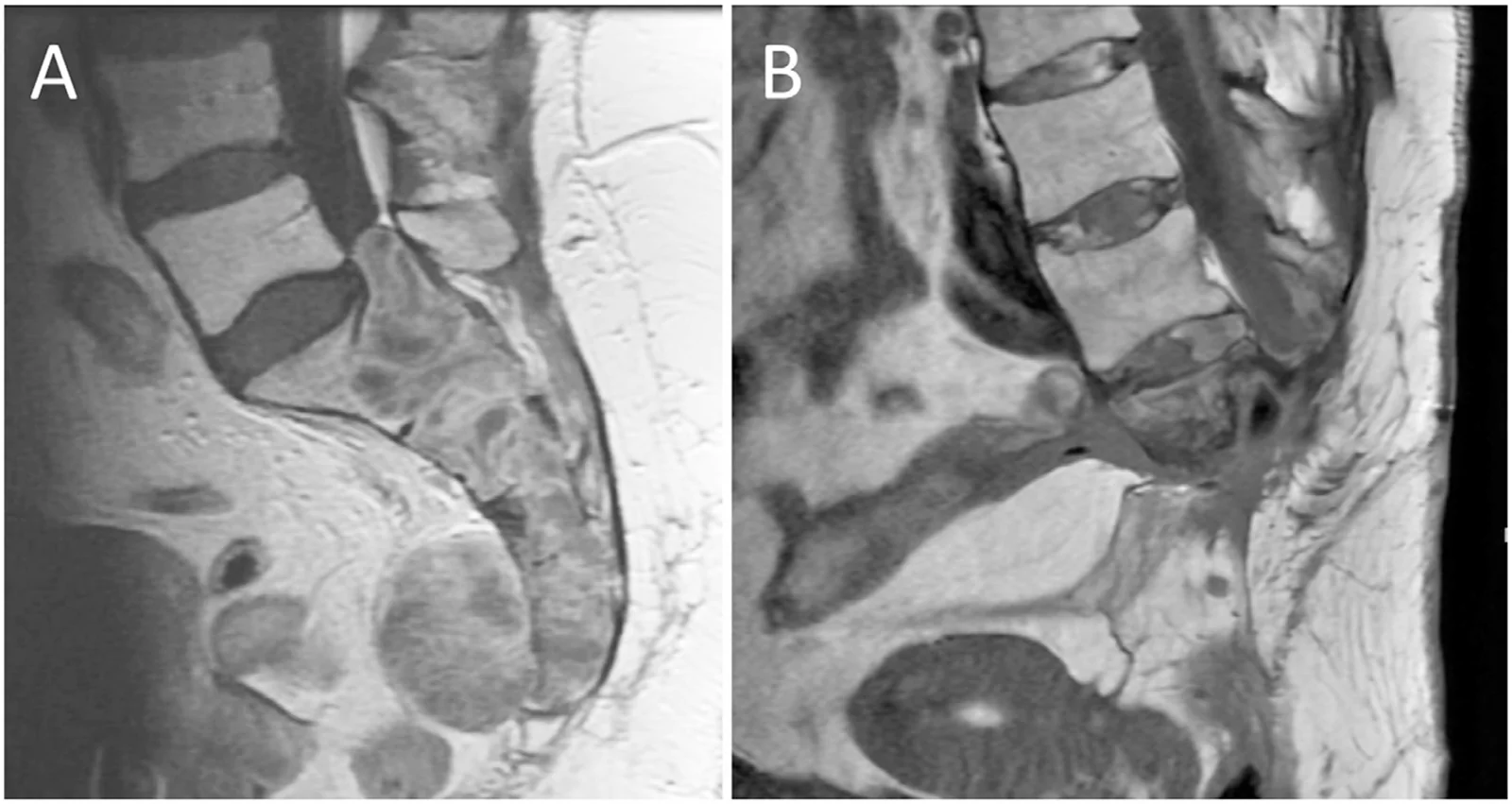
A representative case depicting (A) preoperative and (B) postoperative T1-weighted sagittal magnetic resonance imaging scans of a 49-year-old woman with a high sacral chordoma who underwent neoadjuvant 24-Gy single-fraction stereotactic body radiation therapy and surgical resection with negative margins. She is currently free of disease at 10 years.

Historically, definitive surgery with negative margins was considered the cornerstone of curative treatment of sacral and sacrococcygeal chordoma [4,5]. However, this treatment modality alone has had modest long-term outcomes; representative studies have reported local recurrence (LR) rates of 40% to 46% at 5 years and up to 73% at 10 years after surgical resection, and overall survival (OS) rates of 75% to 77% at 5 years [5-7]. With advances in radiotherapy (RT) techniques, neoadjuvant or postoperative adjuvant RT with surgical resection has been introduced (Fig. 1) and has reduced LR rates and improved OS [8,9]. However, multimodal treatment may be associated with increased rates of complications, given the combined burden of high-dose radiation and an already highly morbid surgical procedure.

More recently, definitive RT using carbon ion, proton, or high-dose hypofractionated photon beam RT to deliver ablative biologically effective doses (BEDs) has been proposed as an option for achieving durable tumor control while avoiding the morbidity of surgical resection and minimizing radiation toxicity [10-13]. Studies have demonstrated that this approach yields LR and OS rates similar to those reported for surgical cohorts but without surgery-related complications [10-13]. Thus, particularly for patients who are poor surgical candidates, have unresectable tumors due to the morbidity related to surgical resection, or find the associated surgical morbidity unacceptable, definitive RT has become a good alternative to surgical resection.

To date, no large single-center studies have directly compared the outcomes following definitive surgery, surgical resection with RT, and definitive RT. Thus, the objective of this study was to compare LR, OS, perioperative outcomes, and complications among patients with primary solitary sacral and sacrococcygeal chordomas treated with these 3 treatment strategies.

## Materials and methods

### Study population and data

We performed a retrospective analysis of patients with sacral or sacrococcygeal chordoma who were treated at a single cancer institution between January 1994 and February 2024. Patients were eligible if they had biopsy-proven primary localized sacral or sacrococcygeal chordomas treated at our institution, or if they were treated for recurrence at our institution and adequate data from the outside institution was available regarding the initial treatment of the primary chordoma. Patients were excluded if they had metastatic disease at presentation, if they had dedifferentiated histology, if a histologic diagnosis could not be ascertained, or if details were unavailable from an outside institution regarding the primary surgical technique, surgical margins, and RT technique.

We retrieved patient, tumor, treatment, and outcome data from electronic medical records. This included information on demographic characteristics (age at diagnosis, age at definitive treatment, sex [as recorded in medical records], race), sacral location of the tumor (high vs. low), treatment modality, presence of dedifferentiated histology, and the type, dose, timing, and fractions of RT received. A high sacral chordoma was defined as an intra- and/or extraosseous tumor extension cranial to the level of the S2-S3 disc space. Additionally, dates of LR, metastatic disease, death, final follow-up, and last new complication were acquired. BED was calculated using an *α/b* ratio of 2 as previously described for chordomas [14,15].

### Outcome measures

The primary outcome measures were LR and OS. Secondary outcome measures were short-term (30-day) and long-term complications. Total complications were defined as the presence of any complication during follow-up, or until patient death. Complications included wound complications (dehiscence, infection, and ulcer), urinary retention, bowel incontinence, venous thromboembolism (VTE), sepsis, urinary tract infection, pneumonia, ureteral transection, bowel perforation, rectal fistula formation, radiculopathy, muscle weakness, chronic osteomyelitis, pathologic sacral fracture, radiation-induced sarcoma, and grade 2 or higher radiation-induced dermatitis. Perioperative and peritreatment outcomes of interest were surgical margins, and 30-day rates of complications, reoperations, and mortality.

### Statistical analysis

We used descriptive statistics to summarize the study cohort and treatment patterns over time. Propensity scores derived via a multinomial logistic regression model that adjusted for sex, age at diagnosis, sacral location (high vs. low), year of diagnosis, and neuroforaminal extension were used to construct inverse probability of treatment weights [16]. The cumulative incidence of LR is reported alongside 95% confidence intervals (CIs) by treatment, for all patients combined, and by margin status among patients who underwent surgery, with Gray’s test applied to compare LR by margin status. LR from end of treatment was modeled using a cause-specific Cox proportional hazards model; LR constituted an event, and patients who were lost to follow-up or died were censored at that time. The model for LR was repeated in a subanalysis of patients who underwent RT to evaluate the association between BED and LR. We modeled OS from end of treatment using a Cox proportional hazards model. The Cox models for LR and OS were weighted by the inverse probability of treatment weights and additionally adjusted for age at diagnosis, sacral location, and the year treatment ended; we report hazard ratios (HRs) and 95% CIs. One patient with missing data on treatment end date was excluded from analyses of LR and OS. Sensitivity analyses for the associations between treatment and LR and OS were performed for patients whose treatment ended in or after 2010 in order to mitigate temporal bias due to potential improvements in treatment techniques over the past 30 years and to ensure more comparable follow-up across the 3 treatment arms. Additionally, we performed a sensitivity analysis restricted to patients with a minimum of 5 years of potential follow-up to ensure a minimum follow-up duration sufficient to capture later events given the indolent nature of many chordomas [17].

The proportions of patients with a complication or reoperation within 30 days are reported and compared across treatment arms using chi-squared tests. The incidence of complications at any time following treatment was modeled using a negative-binomial regression model for count data with an offset of the log of follow-up time for each patient; we report univariable incidence rate ratios (IRR) and CIs. Margin status among patients treated surgically was compared across treatment arms using Fisher’s exact test.

Statistical significance was defined as p<.05. Data analysis was performed using R statistical software (Version 4.3.1).

### Ethical approval

The study was conducted in accordance with the Declaration of Helsinki and received approval from the local institutional review board (protocol #16-1123). Informed consent requirements were waived due to the retrospective and observational nature of the study.

## Results

### Study sample

A total of 113 patients were eligible for and included in the analysis (Table 1). Their mean age was 58 years (standard deviation [SD], 15), and 78 (69%) were male. Forty-nine patients (43%) had a high sacral chordoma. Fifty-five patients (49%) underwent surgery alone, 40 (35%) underwent surgical resection with neoadjuvant or postoperative adjuvant RT, and 18 (16%) received definitive RT. For all patients who underwent surgery, resection was performed with the intent of achieving negative margins. The median follow-up among patients alive at the end of the study was 7.6 years (interquartile range [IQR], 2.7–11 years); it was 8.4 years (IQR, 3.0–14) among patients who had definitive surgery, 7.7 years (IQR, 2.7–9.5) among patients who had surgery with RT, and 3.6 years (IQR, 1.8–5.9) among patients receiving definitive RT. Among the 58 patients who received RT, the most common type was stereotactic body radiation therapy (SBRT) (n=33; 57%), followed by photon-based conventionally fractionated external beam radiation therapy (cEBRT) (n=16; 28%) and proton beam radiation therapy (PBRT) (n=8; 14%) (Table 2). One patient (2%) received an unknown type of adjuvant RT after surgical resection.

### Temporal analysis of treatment patterns

**Fig. 2.**
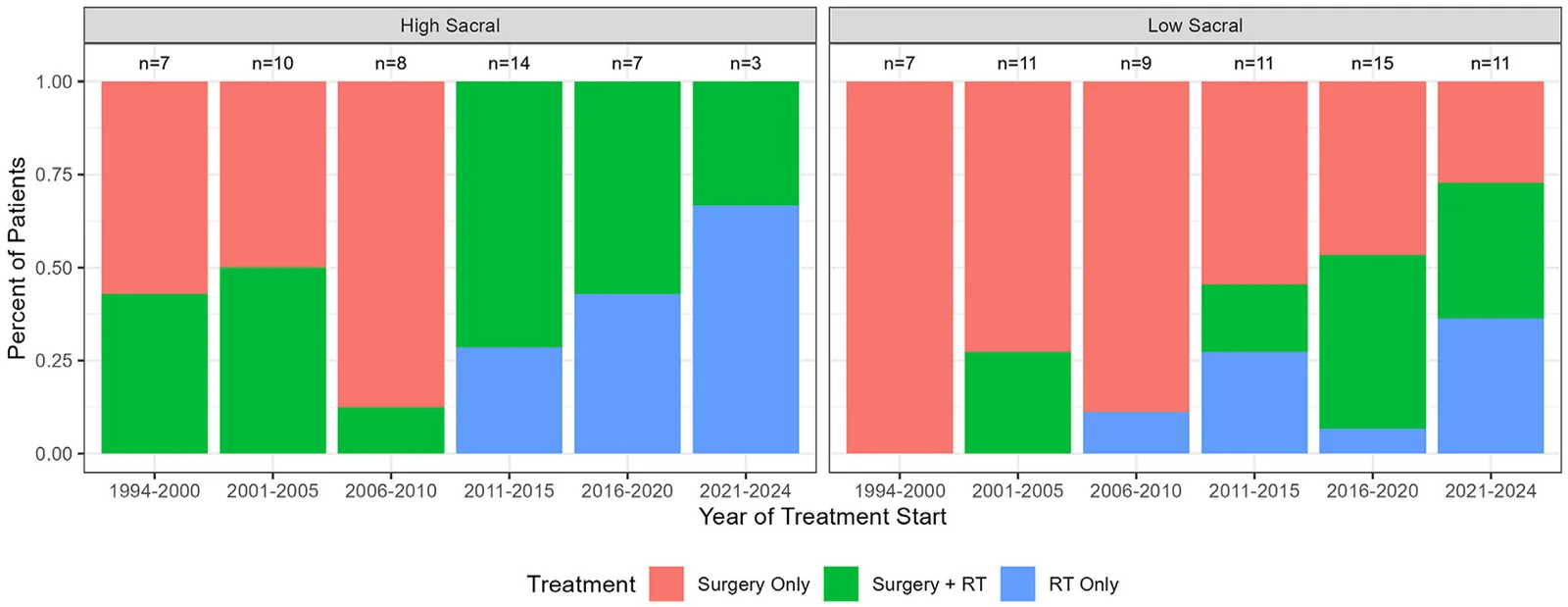
Treatment patterns over time for high sacral (left) and low sacral (right) chordomas.

Treatment patterns for sacral and sacrococcygeal chordoma evolved substantially over the study period, with notable differences by tumor location (Fig. 2). Among patients with high sacral tumors, surgical resection alone was predominant from 1994 to 2010, accounting for the majority of cases, but was replaced by combined modality treatment (surgery + RT) as of 2010. Definitive radiotherapy emerged in 2011 to 2015 and subsequently increased in utilization. Among patients with low sacral tumors, surgery + RT showed moderate uptake beginning in the early 2000s, while definitive RT was increasingly utilized after 2010, with its highest use observed in the most recent cohort (2021–2024). Despite this shift, surgery alone continued to account for a substantial proportion of cases in low sacral disease.

### Local recurrence

**Fig. 3.**
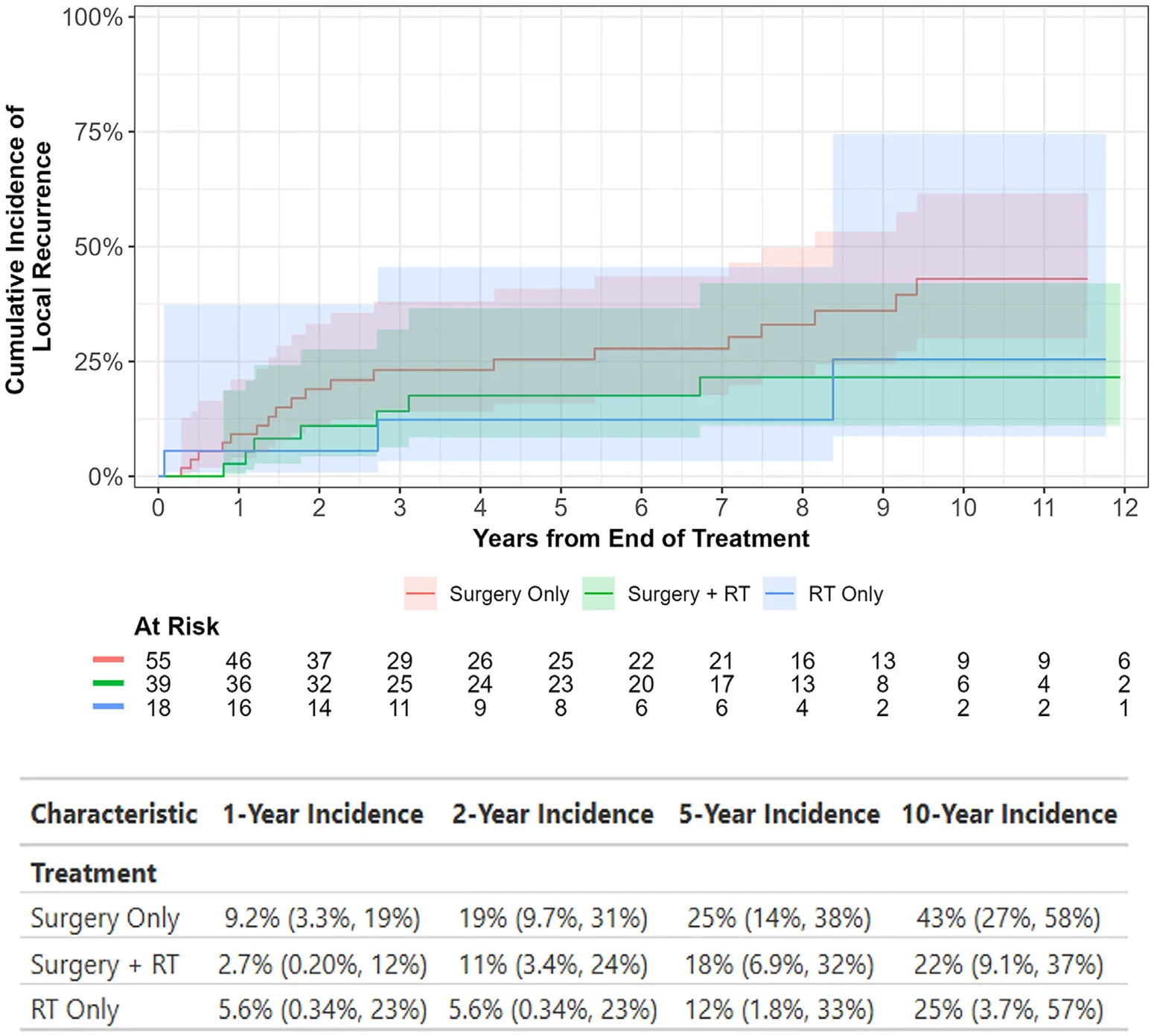
Cumulative incidence of local recurrence by treatment type.

Thirty-one patients had LR, of whom 20 had undergone surgery alone, 8 had undergone surgery with RT, and 3 had received definitive RT. At 5 years, the cumulative incidence of LR was 25% (95% CI, 14%–38%) among patients treated with surgery alone, 18% (95% CI, 6.9%–32%) among those who had surgery with RT, and 12% (95% CI, 1.8%–33%) among patients treated with definitive RT (Fig. 3). In multivariable models, the HRs for LR were 0.48 (95% CI, 0.17–1.31) for surgery with RT and 1.01 (0.25– 4.12) for definitive RT relative to surgery alone, and 2.11 (95% CI, 0.51–8.73) for definitive RT relative to surgery with RT, though the risk of LR did not significantly differ by treatment (p=.3) (Table 3). The results of the sensitivity analyses were consistent with those of the main multivariable model (Table 3).

**Fig. 4.**
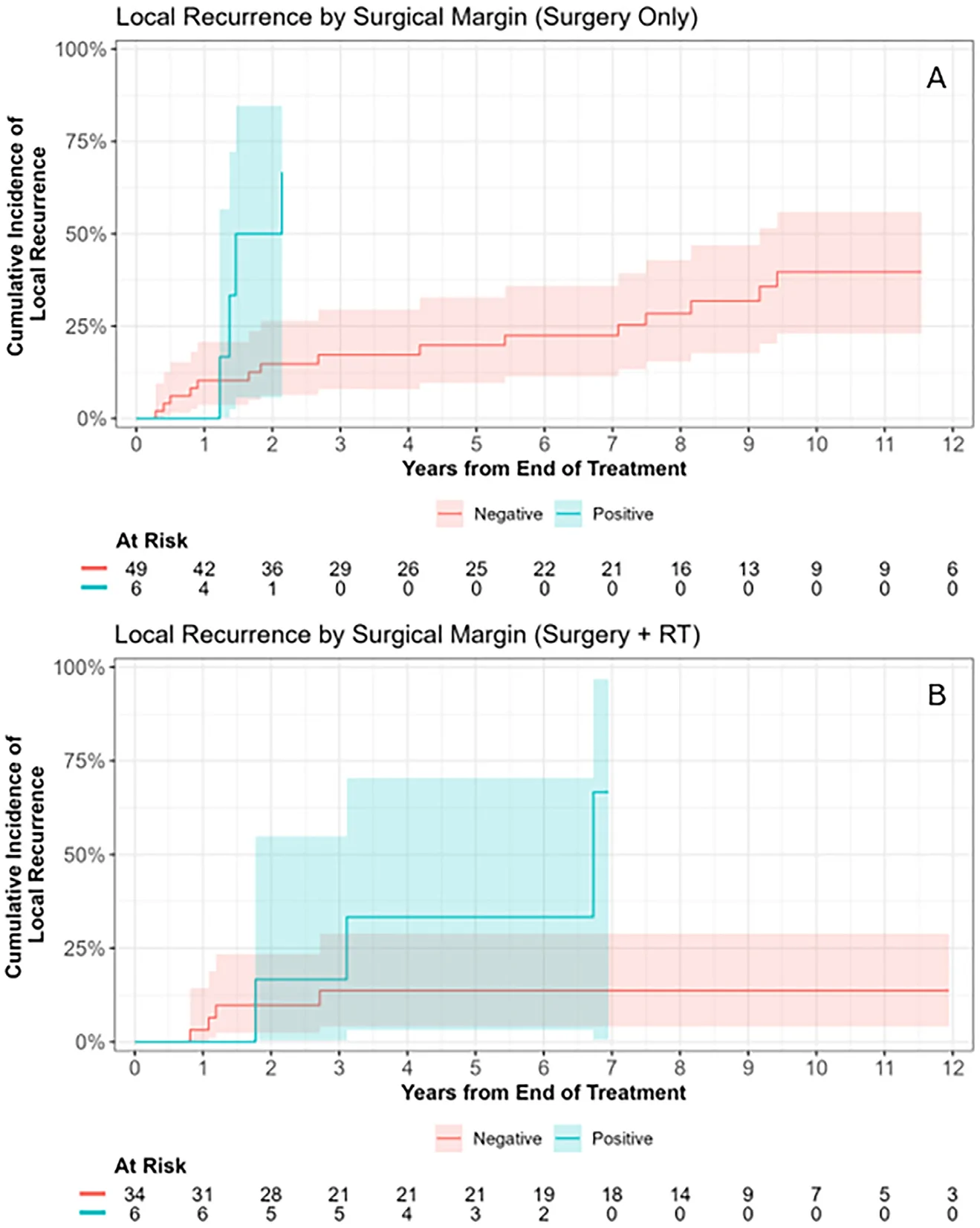
Cumulative incidence of local recurrence by surgical margin status among patients treated with (A) surgery alone and (B) surgery with radiotherapy.

Among the 50 patients who received RT and had BED data available, higher BED was associated with a reduced risk of LR (HR, 0.92; 95% CI, 0.86–0.98; p=.01) (Table 4). Among patients who underwent surgery alone, the risk of LR was higher among those with positive margins than among patients with negative margins (p=.005 in univariable analysis). For example, at 2 years, 50% (95% CI, 5.6%–85%) of patients with positive margins had LR, compared with 15% (95% CI, 6.4%–26%) of those with negative margins (Fig. 4). Small sample sizes and few events (4 among the 6 patients with positive margins and 16 among the 49 patients with negative margins) precluded multivariable analysis. Among patients who underwent surgery with RT, the 5-year incidence of LR was 33% (95% CI, 3.2%–70%) among those with positive margins and 14% (95% CI, 4.2%–29%) among those with negative margins (univariable p=.06; Fig. 4).

### Overall survival

**Fig. 5.**
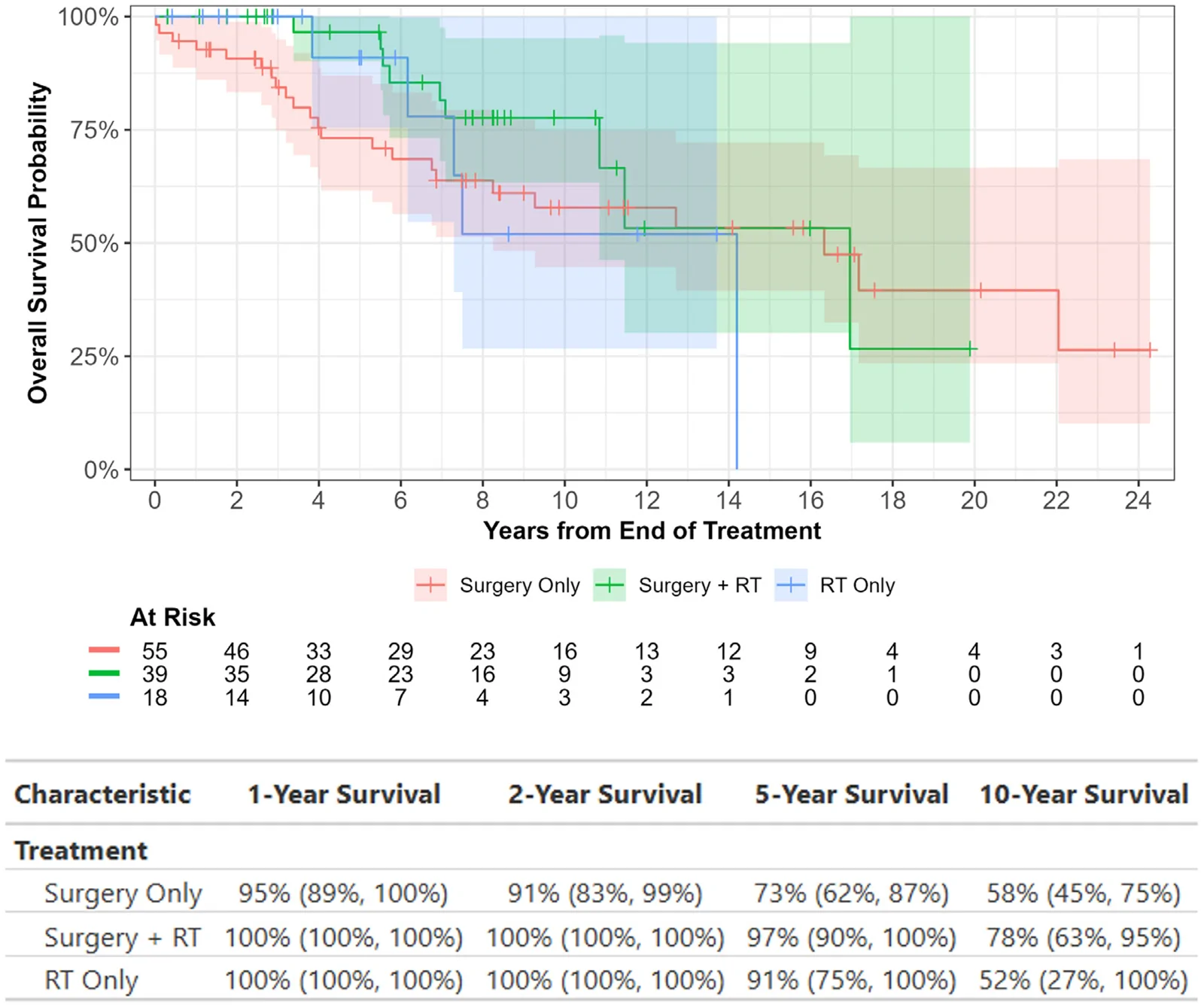
Kaplan–Meier estimates of overall survival by treatment type.

**Fig. 6.**
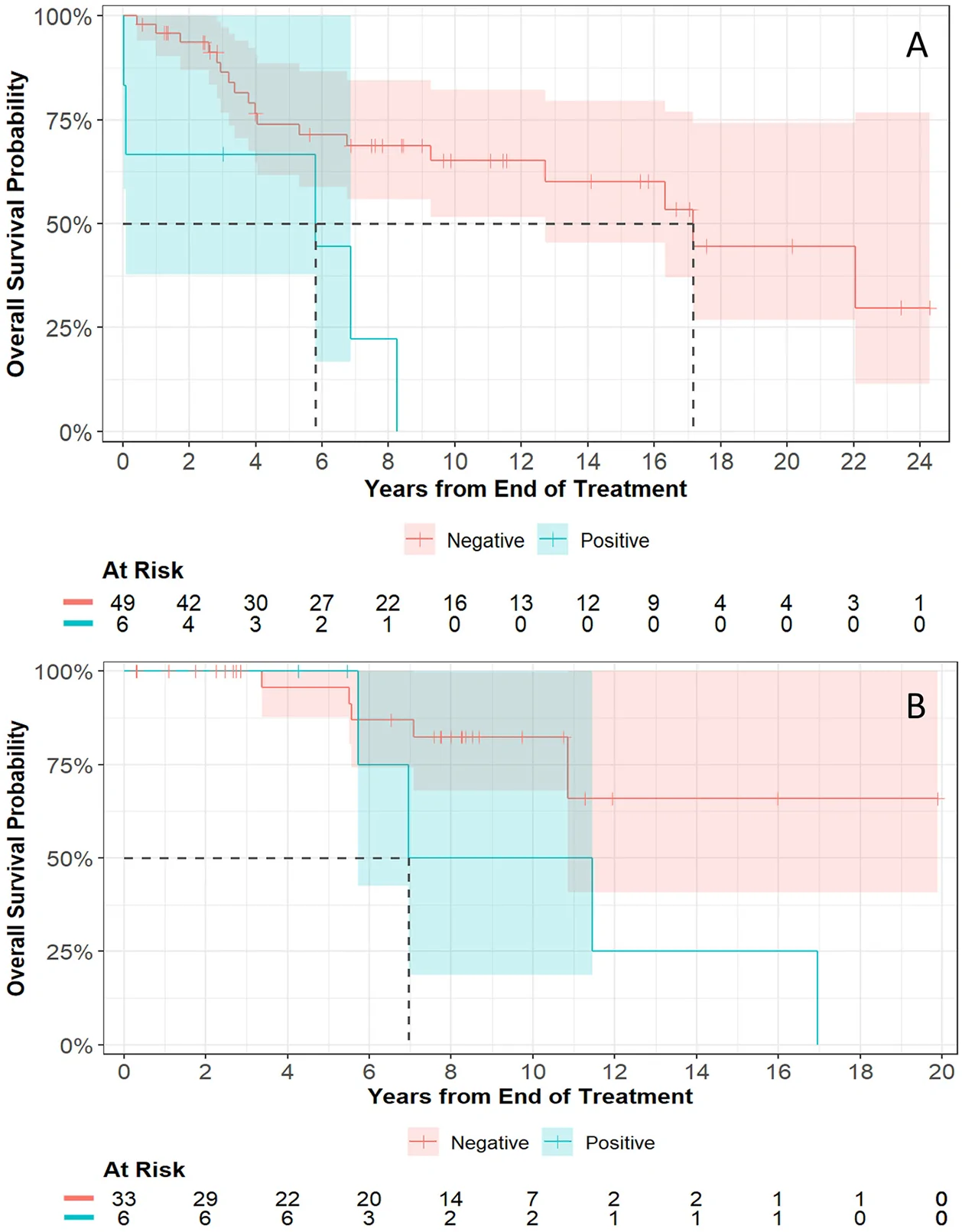
Overall survival by surgical margin status among patients treated with (A) surgery alone or (B) surgery with radiotherapy.

There was a total of 37 deaths (76 patients were alive at end of study or censored at their last visit date); 23 deaths were among patients who underwent definitive surgery, 9 among patients who underwent surgery with RT, and 5 among patients who received definitive RT. Five-year OS was higher among patients who had surgery plus RT (97%; 95% CI, 90%–100%) than among those who had surgery alone (73%; 95% CI, 62%–87%) (Fig. 5). Multivariable analysis demonstrated that OS differed significantly by treatment: the HRs for OS were 0.50 (95% CI, 0.24–1.04) for surgery + RT and 1.86 (95% CI, 0.60–5.79) for RT alone relative to surgery alone, and 3.73 (95% CI, 1.15–2.1) for RT alone relative to surgery + RT. The HRs were generally similar in sensitivity analyses restricted to patients whose treatment began in or after 2010 or who had at least 5 years of potential follow-up and remained significant in the latter group (Table 5). Among patients who had surgery alone, those with positive margins had shorter OS (Fig. 6); however, small sample sizes precluded formal analysis.

### Perioperative/peritreatment outcomes and complications

Negative margins were achieved in 83 (87%) of the 95 patients who underwent surgery without or with RT (Table 6). There were no significant differences between treatment modalities in 30-day reoperation rates (25% for surgery alone vs. 15% for surgery with RT; p=.5). One death occurred within 30 days (surgery alone group). Among the 40 patients who underwent surgery for high sacral chordomas, 34 (85%) had urinary retention and 23 (58%) loss of bowel function at 30 days (not shown). At final follow-up, 34 (85%) had urinary retention and 23 (58%) had loss of bowel function.

The rate of 30-day complications was highest among patients undergoing surgery with RT (34/40; 85%), followed by those undergoing surgery alone (30/55; 55%); it was lowest among patients undergoing definitive RT (1/18; 5.6%). The proportion of patients who had any complication during follow-up was 60% among patients who had surgery alone, 93% among patients who had surgery plus RT, and 56% among patients who underwent definitive RT. In the univariable regression model that accounted for the variation in follow-up times among the treatment modalities, the incidence of any complication during follow-up did not differ by treatment modality; the IRRs were 1.15 (95% CI, 0.58–2.31) for surgery with RT and 0.62 (95% CI, 0.25–1.61) for RT alone relative to surgery alone (p=.4).

## Discussion

The treatment of sacral chordomas poses surgical and clinical challenges distinct from those of treating chordomas of the mobile spine. Over the last 3 decades, treatment approaches have evolved to include 3 main modalities: wide surgical resection alone, surgical resection with RT, and definitive RT. Our analysis of patients seen at a single center across 30 years demonstrates similar LR and OS among the 3 treatment modalities; however, the 30-day complication rates were highest among patients who underwent surgical resection with neoadjuvant or adjuvant RT. Our results suggest that definitive RT may be further explored as a reasonable nonsurgical option, given that it has lower 30-day complication rates; however, longer follow-up is required to determine its durability and late-toxicity profile.

An important observation from this study was that 5-year OS was longer among patients who underwent surgery with an RT regimen than among those who had surgery alone (97% vs. 73%), although the difference was not statistically significant in multivariable analyses, potentially due to the limited sample size and event rate. In addition, we found that among patients who received an RT regimen, treatment with a higher BED was associated with a lower risk of LR. These findings are consistent with recent singlearm and dual-arm studies that have demonstrated that supplementing treatment with high-dose RT is associated with lower incidence of LR and longer OS [8-10]. In an analysis of 76 patients with chordomas of the mobile spine and sacrum, Tobert et al. reported that LR rates were lower and OS was longer if patients treated with surgical resection plus neoadjuvant or adjuvant RT completed the full 70-Gy regimen of PBRT [9]. However, our results contrast with those of Adida et al., who recently reported a 5-year OS rate of only 51% in a series of 24 patients treated with definitive, adjuvant, or salvage SBRT (median BED, 175) for primary or recurrent chordoma [15]. They also found that LR and OS did not differ by patients’ BED exposure (<175 vs. ≥175). The difference between these results and ours may be largely attributable to Adida et al.’s smaller sample size, lower median BED, and inclusion of recurrent chordomas treated with salvage SBRT; most patients in our expanded series received 24-Gy single-fraction SBRT, with a median BED of 312. Thus, the ability of our study to detect a difference in LR suggests that the reduction in LR may only be observed at higher BED levels. Future research to determine a meaningful dichotomy for BED may provide valuable guidance for tailoring treatment to individual patients with sacral chordomas.

Jin et al. analyzed 35 patients with de novo chordomas of the mobile spine or sacrum and found no significant difference in LR or OS between patients receiving definitive SBRT or surgery with SBRT [10]. In addition to high-dose photon RT, both carbon-ion and proton-based RT have demonstrated clinical success in recent years. In a cohort of 67 patients who underwent definitive proton RT at a median total dose of 77.4 Gy, Banfield et al. reported a 5-year OS rate of 83.5% [12]. Yolcu et al. analyzed 911 patients with sacral chordomas treated with carbon-ion RT or surgical resection and found no significant difference in OS but fewer complications among patients undergoing definitive carbon-ion RT [13]. Importantly, the morbidity profile of definitive RT differs substantially from that of surgery, particularly in its delayed presentation. While short-term complication rates were low in our study, clinically significant late toxicities—including radiation-induced radiculopathy, sacral insufficiency fractures, chronic wounds, tumor necrosis with infection, and neurologic decline—may manifest several years after treatment. Given the shorter follow-up in the RT cohort, the true long-term morbidity of this approach may be underestimated. However, these findings suggest that definitive RT may be a reasonable alternative to surgery, not only for patients whose comorbidities or systemic illnesses preclude surgery, but also those with large tumors or high sacral chordomas with inherently higher risks of complications and surgical morbidity.

Over the 3-decade study period, substantial advances occurred in both surgical technique and RT delivery, including the adoption of SBRT and proton therapy. Although we adjusted for treatment year and performed sensitivity analyses, residual confounding related to these temporal changes likely persists. Our findings demonstrated a temporal shift toward increased use of RT-based strategies, particularly definitive RT in the modern era, with the most pronounced change observed in patients with high sacral tumors. This likely reflects evolving RT technologies, increasing experience with high-dose regimens, and greater consideration of treatment-related morbidity associated with extensive sacral resections. In addition, recent data further underscore the evolving role of RT modality selection in chordoma management. A contemporary analysis by El-Hajj et al. demonstrated that proton-based radiotherapy may confer improved survival outcomes compared with photon-based approaches in the adjuvant treatment of chordoma, highlighting the importance of advanced radiation techniques in optimizing disease control [18]. This aligns with growing evidence that dose escalation and conformal delivery techniques are critical determinants of local control, particularly in tumors located near critical neurologic structures where conventional photon therapy may be limited by toxicity constraints. In a large cohort of 101 patients undergoing definitive resection, recurrence occurred in approximately 25% of patients at a mean follow-up of 4 years, with significantly higher risk observed in tumors with a volume of ≥100 cm^3^ (HR, 5.89) and in mobile spine lesions (HR, 7.73), while neoadjuvant radiotherapy was associated with a markedly reduced risk of local recurrence (HR, 0.09). These data highlight that even with aggressive surgical management, tumor biology and burden remain dominant drivers of recurrence, and multi-modality approaches are often required to optimize outcomes [19].

However, it is also important to recognize that high-dose RT carries its own set of unique limitations and potential complications that may warrant a separate discussion during patient counseling regarding treatment options. First, the temporal pattern of complications may differ from that of alternative treatment. Although we found definitive RT to have the lowest long-term complication rate, the median follow-up time in the definitive RT group was only 3.7 years, compared with 6.8 and 7.6 years in the definitive surgery and surgery plus RT groups, respectively. Accordingly, the relatively shorter follow-up in the RT cohort likely underestimates the true burden of RT-related morbidity, and these risks should be considered when counseling patients regarding nonsurgical management. When we accounted for the varying follow-up times in univariable analysis, the incidence of total complications during the follow-up period did not vary significantly by treatment modality, though we are limited by the sample size and event rate per treatment group. We found that complications associated with definitive RT differ from those of the surgical cohorts and occur later. Although the 30-day complication rate was only 5.6% following completion of definitive RT, the rate rose to 56% by the end of follow-up due to the occurrence of such complications as radiation-induced sacral fractures and grade 2 or higher dermatitis. These late complications unique to patients undergoing high-dose RT have been reported elsewhere [20,21]. Thus, patient counseling regarding the different treatment modalities should include the complication profiles and timing associated with each option.

Discussions of surgical morbidity and quality-of-life expectations are important components of preoperative counseling for surgical resection. Due to the necessity of transecting proximal sacral nerve roots, the risks of bowel and bladder function loss are particularly high among patients who undergo resection for high sacral chordomas [22]. Consistent with this, 85% of patients who underwent surgery for high sacral chordomas in our study had loss of bladder function and 58% had loss of bowel function. In addition, prior studies that utilized patient-reported outcome measures to investigate the effect of nerve root transections and level of sacrectomy have demonstrated that patients who have a greater number of proximal nerve root transections and resections have significantly lower quality-of-life scores in physical, mental, and sexual health [11-24]. These findings highlight the importance of detailed counseling and of weighing functional outcomes and long-term quality of life when selecting patients for surgical management, particularly for high sacral chordomas.

Our analysis suggests that margin status is an important prognostic indicator for LR and OS among patients who undergo surgical resection. This is consistent with previous studies that have demonstrated a direct association between positive margins and LR [5,25,26]. In our cohort, positive margins were most commonly seen in cases involving large or high sacral tumors for which attempts to preserve proximal neurologic function or avoid damage to critical organs or neurovascular structures limited the extent of the resection. Our findings further suggest that when negative margins cannot be reasonably obtained due to large tumor size and/or proximity to critical organs and neurovascular structures, either definitive or neoadjuvant/adjuvant RT treatment are important considerations to limit LR. Thus, a multidisciplinary evaluation that ascertains the feasibility of resection and takes into account patient preferences regarding complication profiles is particularly important to the determination of patient-specific treatment.

This study has several important limitations. First, the retrospective, single-institutional design is susceptible to selection bias, residual confounding, and limited generalizability to the broader population. Second, improvements in clinical, surgical, and RT technology and techniques over the 3-decade study period may have introduced temporal bias. However, we mitigated the impact of this bias by including the year of treatment in the multivariable models and performing relevant sensitivity analyses. Third, the amount of follow-up available for the definitive RT group was shorter than that for the surgical groups, which may not be fully corrected for by statistical analysis. Fourth, the small sample in the definitive RT cohort (18 patients) limits the ability to precisely estimate outcomes for this treatment modality and to detect differences in outcomes between treatment modalities. It is also important to note that although we identified high sacral tumors and reported postoperative bowel and bladder dysfunction, we could not uniformly collect sufficiently granular operative data on the exact level and laterality of sacral nerve root sacrifice, including unilateral versus bilateral S2/S3 preservation. Accordingly, we could not evaluate associations between specific nerve root preservation patterns and bowel, bladder, or sexual function. In addition, patient-reported outcomes were not consistently available, which precluded assessment of sexual function and other quality-of-life domains. Within the definitive radiotherapy cohort, we also could not reliably determine whether tumor size, cephalad extent, or proximity to the bowel or thecal sac necessitated compromise in target coverage because of dose constraints. Therefore, we were unable to assess whether such dosimetric limitations were associated with late functional decline or LR. Future studies incorporating detailed operative anatomy, radiation dosimetry, and patient-reported functional outcomes will be necessary to better define these relationships.

Despite these limitations, to our knowledge this is the largest study to date that directly compares the 3 treatment modalities for primary solitary sacral and sacrococcygeal chordoma. The strength of this study was the ability to incorporate 3 decades of techniques and outcomes at a single cancer institution to demonstrate unique clinical considerations when determining the indications for each type of treatment. Our findings support a more individualized, multidisciplinary approach in which each treatment modality is considered for certain patients and the choice depends on multiple factors, including operative risk, comorbidities, tumor location within the sacrum, the surgical morbidity associated with resection, and the patient’s functional expectations.

## Conclusions

Wide surgical resection has been the recommended treatment for solitary primary sacral and sacrococcygeal chordoma. The addition of an RT regimen may improve survival in selected patients. Although definitive RT demonstrated comparable short-term outcomes, longer follow-up is required to determine its durability and late-toxicity profile. Therefore, definitive RT should be considered selectively rather than as a replacement for surgery.

## Figures and Tables

**Table 1:** Characteristics of the study sample

Characteristic	Surgery only (n=55)	Surgery + RT (n=40)	RT only (n=18)	p value
Age at diagnosis (years)	56±16	56±14	68±12	.006*
Follow-up among patients alive at end of study (years)	8.4 (3.0–14)	7.7 (2.7–9.5)	3.6 (1.8–5.9)	na
Male	35 (64)	30 (75)	13 (72)	.5
High sacral chordoma	16 (29)	24 (60)	9 (50)	.009*
Biological effective dose	na	74.0±10	81±9	.01*

Data presented as n (%) except for age (mean ± standard deviation) and follow-up (median [interquartile range]).

* Statistically significant.

RT, radiotherapy; na, not applicable.

**Table 2:** Radiotherapy regimens by treatment group

RT type		Surgery + RT (n=40)	RT only (n=18)
Adjuvant	cEBRT (50.4–80.0 Gy x 28–35)	9 (22.5)	0 (0)
	SBRT (24 Gy x 1)	2 (5.0)	0 (0)
	PBRT (50.4–60 Gy x 28–30)	1 (2.5)	0 (0)
	Unknown	1 (2.5)	0 (0)
Neoadjuvant	cEBRT (50–60 Gy x 25–30)	6 (15.0)	0 (0)
	SBRT (21–36 Gy x 1–3)*	17 (42.5)	0 (0)
	PBRT (50–70 Gy x 25–35)	4 (10.0)	0 (0)
Definitive	cEBRT (64 Gy x 35)	0 (0)	1 (5.6)
	SBRT (18–24 Gy x 1)*	0 (0)	14 (77.8)
	PBRT (70–78 Gy x 34–35)	0 (0)	3 (16.7)

Regimens presented as dose x fractions. Data presented as n (%).

* Most common regimen: 24 Gy x 1 (neoadjuvant, n=16; definitive, n=13).

RT, radiotherapy; cEBRT, conventional external beam radiation therapy; PBRT, proton beam radiation therapy; SBRT, stereotactic body radiation therapy.

**Table 3:** Multivariable analysis of the association between treatment type and local recurrence

Characteristic	All patients (n=112)			Patients treated in/after 2010 (n=61)			Patients with ≥5 years potential follow-up (n=95)		
	No. of events	HR (95% CI)	p value	No. of events	HR (95% CI)	p value	No. of events	HR (95% CI)	p value
Treatment			.3			.7			.3
Surgery only	20	Reference		2	Reference		20	Reference	
Surgery + RT	7	0.48 (0.17–1.31)		2	0.42 (0.04–3.90)		7	0.47 (0.17. 1.29)	
RT only	3	1.01 (0.25–4.12)		3	0.60 (0.06–6.61)		3	1.00 (0.25. 3.96)	
Age at diagnosis	30	1.07 (0.82–1.40)	.6	7	1.35 (0.67–2.74)	.4	30	1.07 (0.82. 1.39)	.6
Sacral location			.13			.5			.14
High	16	Reference		3	Reference		16	Reference	
Low	14	0.51 (0.21–1.23)		4	1.83 (0.38–8.80)		14	0.52 (0.22. 1.23 )	
Year of treatment	30	0.89 (0.84–0.94)	<.001*	7	0.76 (0.59–0.98)	.033	30	0.89 (0.84. 0.95 )	<.001*

* Statistically significant.

HR, hazard ratio; CI, confidence interval; RT, radiotherapy.

**Table 4:** Multivariable analysis of the association between biologically effective dose and local recurrence, among patients treated with radiation therapy

Characteristic	No. of events	HR (95% CI)	p value
BED	5	0.92 (0.86–0.98)	.011*
Treatment			.09
RT only	3	Reference	
Neoadjuvant RT + surgery	1	0.14 (0.02–0.88)	
Surgery + adjuvant RT	1	0.42 (0.06–2.97)	
Sacral location			
High	3	Reference	.3
Low	2	0.36 (0.06–2.31)	

* Statistically significant.

HR, hazard ratio; CI, confidence interval; RT, radiotherapy.

**Table 5:** Multivariable analysis of the relationship between treatment type and overall survival

	All patients (N=112)			2010 + (N=61)			≥ 5 years potential follow-up (N=95)		
Variable	No. of events	HR (95% CI)	p value	No. of events	HR (95% CI)	p value	No. of events	HR (95% CI)	p value
Treatment			.047			.3			.050*
Surgery only	23	Reference		1	Reference		23	Reference	
Surgery + RT	9	0.50 (0.24–1.04)		1	0.12 (0.01–1.80)		9	0.50 (0.24–1.04)	
RT only	5	1.86 (0.60–5.79)		5	1.18 (0.20–7.07)		5	1.84 (0.59–5.71)	
Age at diagnosis	37	1.87 (1.26–2.78)	.002*	7	2.09 (0.84–5.23)	.11	37	1.86 (1.25–2.78)	.002*
Sacral location			.007*			.7			.007*
High	21	Reference		2	Reference		21	Reference	
Low	16	0.42 (0.23–0.79)		5	1.49 (0.22–10.3)		16	0.42 (0.23–0.79)	
Year of treatment	37	0.86 (0.81–0.92)	<.001*	7	1.25 (0.89–1.76)	.2	37	0.86 (0.81–0.92)	<.001*

* Statistically significant.

HR, hazard ratio; CI, confidence interval; RT, radiotherapy.

**Table 6:** Perioperative/peritreatment outcomes and total complications at final follow-up

Characteristic	Surgery only (n = 55)	Surgery + RT (n = 40)	RT only (n = 18)	p value
Negative margins	49 (89)	34 (85)	na	.8
30-day reoperation	14 (25)	6 (15)	na	.5
30-day mortality	1 (1.8)	0 (0.0)	0 (0.0)	>.9
30-day complications	30 (55)	34 (85)	1 (5.6)	<.001*
Wound complication	12 (22)	5 (13)	0 (0.0)	
Urinary retention	15 (27)	29 (73)	0 (0.0)	
Bowel incontinence	9 (16)	20 (50)	0 (0.0)	
Venous thromboembolism (DVT/PE)	1 (1.8)	2 (5.0)	0 (0.0)	
Sepsis	0 (0.0)	2 (5.0)	0 (0.0)	
Urinary tract infection	2 (3.6)	2 (5.0)	0 (0.0)	
Pneumonia	1 (1.8)	1 (2.5)	0 (0.0)	
Ureteral transection	1 (1.8)	0 (0.0)	0 (0.0)	
Radiculopathy	0 (0.0)	1 (2.5)	1 (5.6)	
Muscle weakness	1 (1.8)	1 (2.5)	1 (5.6)	
Total complications	30 (55)	34 (85)	10 (56)	.4
Wound complication	12 (22)	6 (15)	2 (11)	
Urinary retention	15 (27)	29 (85)	3 (17)	
Bowel incontinence	9 (16)	20 (50)	1 (5.6)	
Venous thromboembolism (DVT/PE)	2 (3.6)	2 (5.0)	0 (0.0)	
Sepsis	0 (0.0)	2 (5.0)	0 (0.0)	
Urinary tract infection	2 (3.6)	2 (5.0)	0 (0.0)	
Pneumonia	1 (1.8)	1 (2.5)	0 (0.0)	
Ureteral transection	1 (1.8)	0 (0.0)	0 (0.0)	
Radiculopathy	0 (0.0)	1 (2.5)	1 (5.6)	
Muscle weakness	1 (1.8)	1 (2.5)	1 (5.6)	
Bowel perforation	1 (1.8)	0 (0.0)	0 (0.0)	
Rectal fistula	0 (0.0)	1 (2.5)	0 (0.0)	
Chronic osteomyelitis	0 (0.0)	0 (0.0)	1 (5.6)	
Sacral fracture	0 (0.0)	1 (2.5)	3 (16.7)	
Radiation-induced sarcoma	0 (0.0)	1 (2.5)	0 (0.0)	
Radiation-induced dermatitis	0 (0.0)	0 (0.0)	2 (11.1)	

Data presented as n (%).

* Statistically significant.

RT, radiotherapy; na, not applicable; DVT, deep vein thrombosis; PE, pulmonary embolism.
